# Abnormal Contextual Modulation of Visual Contour Detection in Patients with Schizophrenia

**DOI:** 10.1371/journal.pone.0068090

**Published:** 2013-06-18

**Authors:** Michael-Paul Schallmo, Scott R. Sponheim, Cheryl A. Olman

**Affiliations:** 1 Graduate Program in Neuroscience, University of Minnesota, Minneapolis, Minnesota, United States of America; 2 Veterans Affairs Medical Center, Minneapolis, Minnesota, United States of America; 3 Department of Psychiatry, University of Minnesota, Minneapolis, Minnesota, United States of America; 4 Department of Psychology, University of Minnesota, Minneapolis, Minnesota, United States of America; Ecole Polytechnique Federale de Lausanne, Switzerland

## Abstract

Schizophrenia patients demonstrate perceptual deficits consistent with broad dysfunction in visual context processing. These include poor integration of segments forming visual contours, and reduced visual contrast effects (e.g. weaker orientation-dependent surround suppression, ODSS). Background image context can influence contour perception, as stimuli near the contour affect detection accuracy. Because of ODSS, this contextual modulation depends on the relative orientation between the contour and flanking elements, with parallel flankers impairing contour perception. However in schizophrenia, the impact of abnormal ODSS during contour perception is not clear. It is also unknown whether deficient contour perception marks genetic liability for schizophrenia, or is strictly associated with clinical expression of this disorder. We examined contour detection in 25 adults with schizophrenia, 13 unaffected first-degree biological relatives of schizophrenia patients, and 28 healthy controls. Subjects performed a psychophysics experiment designed to quantify the effect of flanker orientation during contour detection. Overall, patients with schizophrenia showed poorer contour detection performance than relatives or controls. Parallel flankers suppressed and orthogonal flankers enhanced contour detection performance for all groups, but parallel suppression was relatively weaker for schizophrenia patients than healthy controls. Relatives of patients showed equivalent performance with controls. Computational modeling suggested that abnormal contextual modulation in schizophrenia may be explained by suppression that is more broadly tuned for orientation. Abnormal flanker suppression in schizophrenia is consistent with weaker ODSS and/or broader orientation tuning. This work provides the first evidence that such perceptual abnormalities may not be associated with a genetic liability for schizophrenia.

## Introduction

In everyday visual perception, objects and paths are defined by visual contours. Contour detection is a perceptual process that is critical for identifying visual edges and boundaries, plays an important role in figure-ground segmentation, and is essential for locating and recognizing objects (for a review, see [1]). Contours are sometimes composed of individual elements that are spatially separated, for example when one object occludes part of another. When broken contours are encountered during normal vision, attributes or features such as the contrast, spacing, and relative orientation of contour elements, as well as curvature, closure, and contour length strongly influence perception, as demonstrated in psychophysical [2–4], electrophysiological [5], and neuroimaging experiments [6]. These observations are in general agreement with the rules of perceptual organization, such as proximity, continuity and similarity, as described in Gestalt psychology [7]. Patients with schizophrenia perform worse than healthy controls in contour integration paradigms [8–13], but the manner in which stimulus features affect this perceptual deficit is not fully understood.

Not only do the features of a visual contour affect perception, but the context (or background) in which a contour appears also modulates perceptual saliency, an effect commonly referred to as contextual modulation. Orientation-dependent surround suppression (ODSS) is one form of contextual modulation that applies to a broad range of stimuli; the perceptibility of a target is affected by the position and relative orientation of nearby stimuli. Recent psychophysical work in the field of contour perception has shown that parallel flanking elements tend to suppress perception of target contours, while orthogonal flankers cause less suppression, in agreement with ODSS [14–17]. Extensive investigations of contextual effects during early visual processing have been made among healthy subjects in past decades, and several groups have recently demonstrated weaker surround suppression effects among patients with schizophrenia compared with controls [18–22]; but see [23,24].

Both contour integration and surround suppression have been highlighted as examples of well documented visual abnormalities in schizophrenia whose investigation may provide insight into the neural underpinnings of this disorder [25]. However, it is not yet known to what extent genetic liability for schizophrenia may contribute to such abnormalities. Further, previous investigations of contour integration deficits in schizophrenia have not specifically examined the role of surrounding stimulus orientation during task performance. Thus, it is not clear how surround suppression deficits in schizophrenia may affect contour perception. In order to better understand the neural mechanism(s) underlying impaired contour detection and abnormal ODSS in this disorder, we examined the performance of patients with schizophrenia, unaffected first-degree biological relatives of schizophrenia patients, and healthy controls during a contour detection paradigm, while manipulating the local orientation context in which target contours appeared. Computational modeling allowed us to separately and quantitatively characterize baseline task performance, the strength of contextual modulation, and its dependence on flanker orientation.

## Methods

## Summary

**Figure 1:**
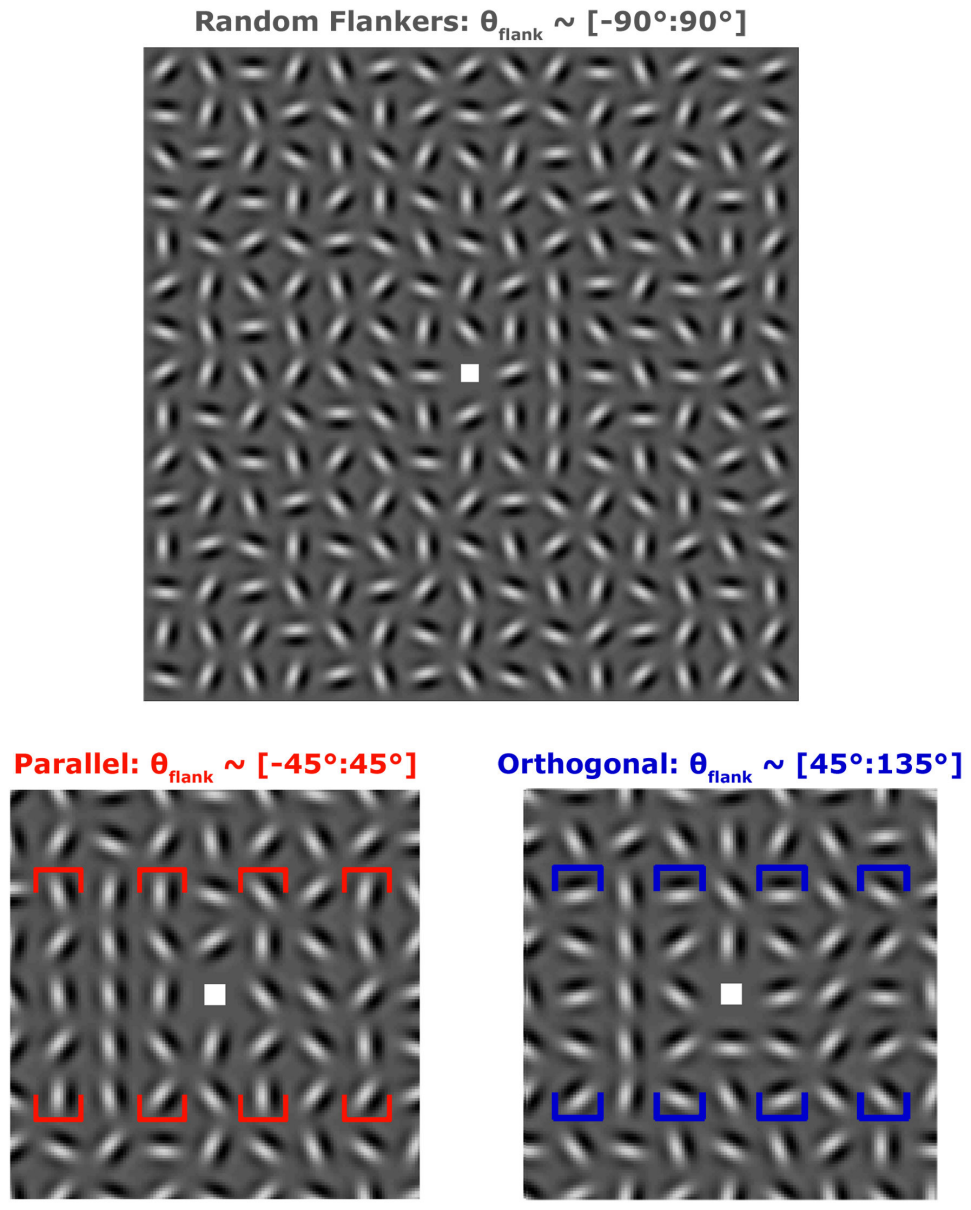
Example Stimuli. Top, Random condition. Target contours (composed of 5 Gabors) were presented in a vertical line in the second column to either the right (as shown in top example) or left of fixation. The Gabors horizontally adjacent to possible target positions are termed flankers, and were oriented randomly in this condition. The distribution of flanker orientations is shown at the top of each panel in brackets. Bottom left, zoomed region to show detail of Parallel condition. Average orientation of flankers (four sets bracketed in red) is parallel to the vertical contour axis. Target contour is presented on the left in both bottom panels. Flankers surrounded both possible target contour locations on every trial. Bottom right, detail of Orthogonal condition. Flankers (bracketed in blue) are on average oriented orthogonal to the vertical target contour.

Patients with schizophrenia, unaffected first-degree biological relatives of schizophrenia patients, and healthy control subjects were recruited to perform a contour detection task. Subjects detected an open vertical contour within a briefly presented array of Gabor stimuli (Gaussian-enveloped sinusoidal luminance modulation, see Figure 1). Target contours were presented either to the right or to the left of fixation and were flanked by Gabors that were parallel, orthogonal or randomly oriented relative to the vertical contour, which defined the stimulus condition. Our group has previously examined contour detection in healthy adults using this paradigm [15]. Task performance was quantified in terms of contour detection thresholds corresponding to the level of orientation jitter for which a subject would detect target contours with 79% accuracy.

### Participants

Twenty eight outpatients (25 with schizophrenia, 3 with schizoaffective disorder – depressed type), 15 first-degree biological relatives of schizophrenia patients, and 29 healthy controls were recruited through the VA Hospital in Minneapolis, MN. Participants were excluded according to the following criteria: English as a second language, mental retardation, current alcohol abuse/drug dependence, current or past central nervous system condition, history of head injury with skull fracture or substantial loss of consciousness, history of electroconvulsive therapy, age less than 18 or greater than 60. Healthy controls were absent diagnoses of bipolar disorder and any psychotic disorder in themselves and their first-degree biological relatives.

The Structured Clinical Interview for DSM Disorders and the Psychosis Module of the Diagnostic Interview for Genetic Studies [26] were completed with each participant, and DSM-IV-TR diagnoses [27] were made by a doctoral-level clinical psychologist. All participants had psychiatric functioning assessed using the Brief Psychiatric Rating Scale (BPRS) [28], with controls and relatives additionally completing the Schizotypal Personality Questionnaire (SPQ) [29]. IQ was estimated from the Wechsler Adult Intelligence Scale (WAIS-III) [30]. Parental education was assessed among patients and controls using a 7 point scale self-report questionnaire, with 1 corresponding to completing 7^th^ grade or less, and 7 indicating completion of a graduate degree. Mean education score from both parents was taken for each subject. Medication dosages for schizophrenia patients were converted to Chlorpromazine equivalents (in milligrams) [31]. Demographic data are presented in Table 1. Age, parental education, and SPQ scores were not different between groups. Gender distributions differed, with more males than females recruited among patients. BPRS scores were higher, and years of education were lower for patients and relatives than for controls. Estimated IQ was marginally different between groups, and tended to be lower among patients than controls. All participants had normal or corrected-to-normal vision.

**Table 1:** Subject Group Demographics.

**Index**	**Scz** (*n* = 25)	**Rel** (*n* = 13)	**Cont** (*n* = 28)	**Statistics**
Age (years)	41.8 (11.9)	40.1 (14.7)	45.1 (11.6)	F_(2,63)_ = 0.88, *p* = 0.42
Gender (*n*)^1^				Χ^2^_(2)_ = 8.24, *p* = 0.02
Male	4	7	14	
Female	21	6	14	
Education (years)^2^	13.5 (1.5)	13.2 (1.4)	15.2 (1.8)	F_(2,63)_ = 9.83, *p* < 0.01
Estimated IQ^3^ (from WAIS-III)	97.8 (17.6)	107 (17.2)	109 (12.2)	F_(2,57)_ = 3.15, *p* = 0.05
Parental Education^a^	4.8 (1.2)	NA	5.0 (1.12)	F_(1,51)_ = 0.48, *p* = 0.49
Overall Symptomatology^4^ (BPRS Total Score)	42.7 (13.1)	31.9 (6.4)	27.6 (4.02)	F_(2,61)_ = 18.5, *p* < 0.01
Schizotypal Characteristics (SPQ Total Score)	NA	15.5 (13.9)	9.7 (6.52)	F_(1,29)_ = 2.61, *p* = 0.12
CPZ Equivalents^b^	270 (219)	NA	NA	NA

All data are presented as Mean (Standard Deviation), unless otherwise noted.

Cont = healthy control group, Rel = first-degree relative group, Scz = schizophrenia patient group. WAIS-III – Wechsler Adult Intelligence Scale, 3rd Edition. BPRS = 24-item Brief Psychiatric Rating Scale. SPQ = Schizotypal Personality Questionnaire. NA = not applicable.

a Parental education was assessed using a self-report questionnaire on a 7 point rating scale (see Methods).

b Medication dosages were calculated in Chlorpromazine equivalents (mg).

1 There was a marginally significant difference in gender distribution between patients and healthy controls following Yates’s and Bonferroni corrections, Χ^2^_(1)_ = 5.37, p 0.06.

2 Education was significantly higher among the healthy control group compared with both patients and first-degree relatives, Tukey’s HSD, *p* < 0.05.

3 There was a marginally significant difference between the Estimated IQ scores for patients and healthy controls, Tukey’s HSD, p 0.05.

4 BPRS scores were significantly lower among the healthy control group compared with both patients and first-degree relatives, Tukey’s HSD, *p* < 0.05.

Estimated IQ data were not obtained for 6 healthy controls subjects. One first-degree relative did not report parental education. One healthy control and one first-degree relative did not complete the BPRS. Eight healthy controls and two first-degree relatives did not complete the SPQ.

### Ethics Statement

Experimental protocols were approved by Institutional Review Boards at the University of Minnesota and Minneapolis VA Medical Center. Subjects gave written informed consent prior to participation, and were paid $15 per hour. The researcher conducting the consent process provided a description of the protocol, outlined the potential risks and benefits of participation, and explained that the decision to participate had no bearing on services obtained at the VA Medical Center, including psychiatric treatment for patients, as stated in the consent form. Individuals who declined to participate in the study were not disadvantaged in any way.

### Stimuli

Stimuli were presented using MATLAB (The MathWorks) and Psychtoolbox [32,33] software on a MacMini running OSX. Images were displayed on a Dell 19″ monitor that subtended 35.1 x 26.7 degrees of visual angle at a viewing distance of 61 cm. Monitor color look-up table was linearized using custom software. Stimuli consisted of Gabor patches in grids of 15 x 15 elements. Grids subtended 12°. Vertical target contours comprised five aligned Gabors of the same spatial phase located at 1.6° eccentricity to the left or right of the central fixation square. Each Gabor consisted of a 2 cycles per degree sine wave grating modulated by a Gaussian envelope (σ = 0.17°), spaced 0.8° from one another (i.e., 1.6λ separation, where λ is the wavelength of the sine wave grating). Spacing, carrier frequency and eccentricity were selected to maximize flanker orientation effects based our previous work [15]. Gabors were presented at 80% contrast. Background was set to mean gray. Orientation of non-target, non-flanker Gabors was random, but differed by at least 30° between cardinal neighbors to prevent perception of an unintended contour.

Contour detection thresholds were measured for three conditions. In the Random condition, Gabors immediately to the right and left (termed flankers) of the contour were randomly oriented (Figure 1, top). In the Parallel and Orthogonal conditions, flanker orientation was drawn from a uniform distribution with ± 45° range centered on 0° or 90° relative to the vertical contour, respectively (flankers bracketed in Figure 1, bottom). Note that the orientation of flankers adjacent to both possible target positions (left and right) was drawn from the flanker distribution, regardless of target location on a given trial. This was done to prevent flanker orientation from providing a cue for target detection.

### Procedure

Subjects were instructed to use their peripheral vision to detect a contour either to the right or to the left of fixation, and to press the corresponding arrow key. Task difficulty was controlled by varying orientation jitter within the contour. Relative orientation of contour elements was adjusted in steps of 4.5° of jitter (range 0–45°). Jitter increased after three consecutive correct responses, and decreased after one incorrect response. This 3-up 1-down staircase converges on a contour detection threshold at the jitter level for which targets are detected with 79% accuracy [34]. The task was organized into blocks of 30 trials. Flanker orientation was held constant within blocks. Subjects completed at least 3 blocks per condition (9 total). The order of blocks was pseudo-randomized.

At trial onset, the fixation mark appeared for 500 msec. Stimuli were then presented for 150 msec. Response time was not limited. Feedback was given for 100 msec after each response. The fixation mark turned green after correct or red after incorrect responses, then disappeared for 500 msec. Total minimum inter-stimulus interval was 1.1 sec. At next trial onset, the fixation mark (and subsequent stimulus array) was randomly moved within 0.5° of the center of the screen. This eliminated the possibility of successfully performing the task by fixating on potential target positions. Fixation and target positions never overlapped in sequential trials. Task performance was monitored by research staff. Reaction times (RTs) were measured for each trial. Median RTs within blocks were compared between groups in a repeated measures analysis of variance, with subjects nested within groups (abbreviated ANOVA in Results). RTs were not different between groups (*F*_(2,64)_ = 0.89, *p* = 0.42). Median RT across subjects was 633 msec.

Prior to the beginning of main experiment, subjects saw three example stimuli sequentially to ensure task comprehension. In these examples, jitter increased from 0° to 13.5°. Subjects next practiced the task before data collection. Practice consisted of at least two sets of 8 trials, continuing until subjects achieved > 80% accuracy. Contour jitter during the two practice sessions was 0° and 4.5°, respectively. Flanker orientation during practice was random.

### Analysis

Contour detection thresholds were obtained for each block of 30 trials by taking the mean jitter level of the last 3 trials in each block. Thresholds were not calculated if the jitter value was 0° for more than 3 of the last 5 trials in a block, as this indicated performance at floor level. Thresholds were also not calculated if the standard deviation of the jitter values during the last 5 trials was greater than 3.5°, because this indicated unstable performance at the end of the block, which would produce an unreliable threshold estimate. Of 765 runs in all subjects, 39 thresholds were not estimated due to floor performance, and 105 were not estimated due to poor convergence. The distributions of excluded runs did not differ between groups (Χ^2^_(2)_ values < 4.44, *p* values > 0.10). Subjects were excluded from further analyses if they had fewer than 5 total threshold estimates, or zero thresholds in any condition. Data from 3 patients with schizophrenia, 2 relatives of schizophrenia patients, and 1 healthy control were excluded in this way. The observed pattern of results was not altered by data exclusion. Data from 25 schizophrenia patients, 13 relatives, and 28 controls were included in the final analyses. Contextual modulation indices were calculated for Parallel and Orthogonal blocks for each subject by taking contour detection thresholds and subtracting the subject’s mean Random condition threshold. When appropriate, *p* values were corrected for multiple comparisons using Tukey’s Honestly Significant Difference (HSD) or False Discovery Rate (FDR) corrections.

### Computational Modeling

The following model was used to characterize the dependence of task performance on flanker orientation:

$$T=T_{0}+c_{s}{\text{N}}_{s}(\theta_{rel}|\;0{}^{\circ},\;\sigma {\;}_{s}^{2})$$ (1)

where *T* is the observer’s contour detection threshold, and *T*_0_ sets the performance baseline. The term *c*_*s*_ scales the amplitude of flanker suppression, which is defined by N_*s*_, a circular normal function:

$${Ν}_{s}(\theta_{rel}|\;\;\mu ,\;\sigma {\;}_{s}^{2})=e^{\frac{\cos(\theta_{rel}-\mu )\;-1}{\sigma {\;}_{s}^{2}}}$$ (2)

Using a mean (*μ*) of 0°, its magnitude is a function of average flanker orientation relative to the contour (θ_*rel*_ = 0, 45°, or 90°), and its orientation tuning width is set by σ_*s*_. Contour detection thresholds from all three flanker conditions for schizophrenia patients and healthy controls were fit with this model using MATLAB’s *lsqcurvefit* function. *T*_0_ was constrained within the interquartile range of patient and control Orthogonal thresholds, *c*_*s*_ was constrained between 0 and the (negative) upper limit of *T*_0_, and σ_*s*_ between 0 and 360°. Statistical significance for parameter differences between groups was assessed using a bootstrap procedure, resampling subject data within groups with replacement across 2000 iterations.

## Results

### Contour Detection Performance

**Figure 2:**
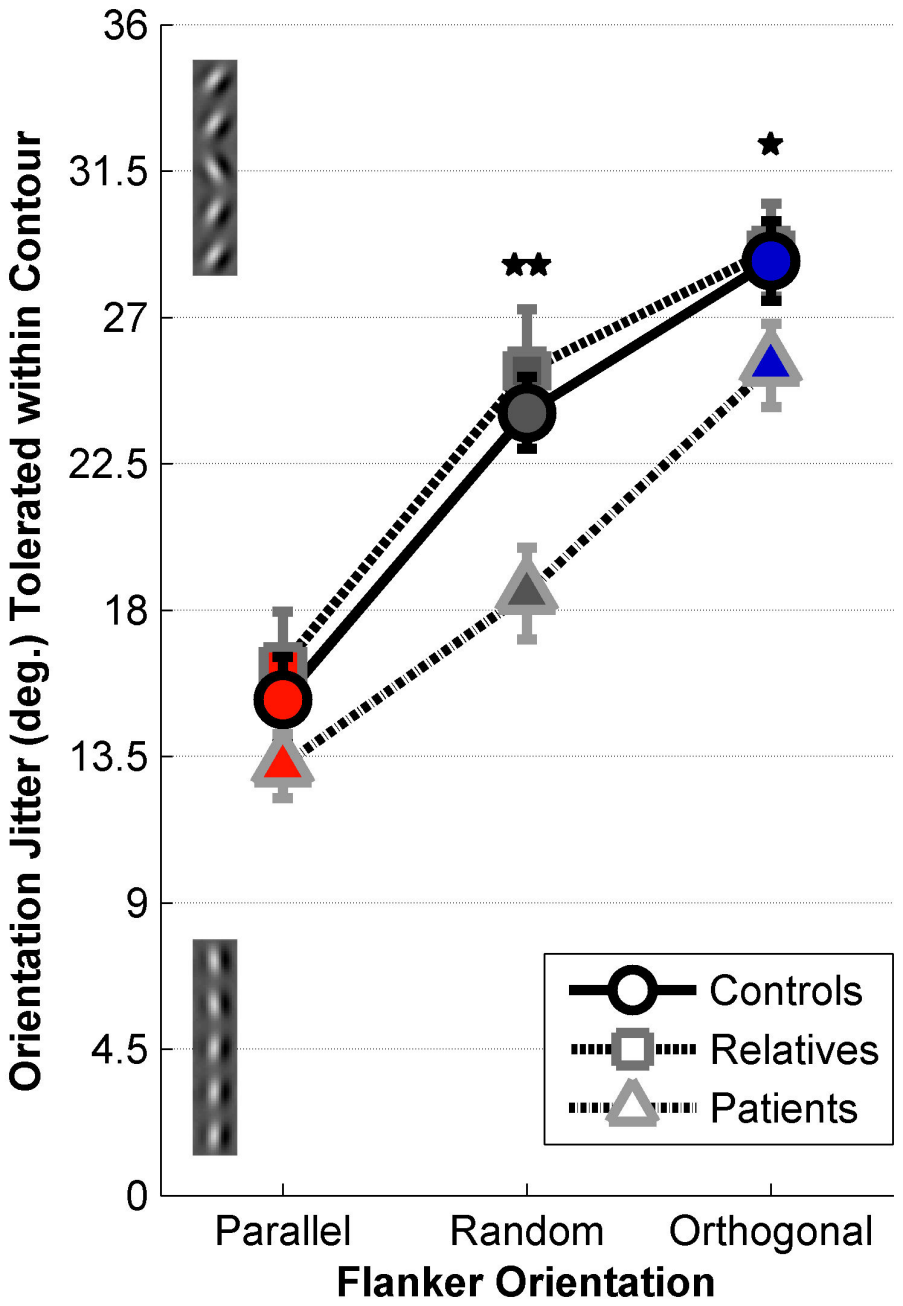
Contour Detection Thresholds. Mean contour detection thresholds are plotted for 28 healthy controls (circles), 13 first-degree relatives (squares), and 25 patients with schizophrenia (triangles) for the Parallel (red), Random (gray), and Orthogonal (blue) conditions. Example contours with 4.5° jitter (bottom) and 31.5° jitter (top) are shown along the y-axis. Error bars are S.E.M. Double asterisk indicates significant differences in Random condition thresholds between schizophrenia patients and both healthy controls and first-degree relatives, single asterisk indicates a significant difference in Orthogonal condition thresholds between patients and relatives. Corrected for multiple comparisons via Tukey’s HSD, *p* < 0.05.

Contour detection thresholds were examined across three flanker orientation conditions (Parallel, Random, and Orthogonal) and between subject groups (schizophrenia patients, first-degree relatives, and healthy controls). Higher thresholds indicated more tolerance for orientation jitter within the contour, and thus better contour detection performance. We observed a significant main effect of condition (ANOVA, *F*_(2,63)_ = 145, *p* < 0.001); contour detection performance was worst in the presence of parallel flankers and best in the presence of orthogonal flankers (Figure 2). This is consistent with previous reports [14–16]. We also observed a significant main effect of group (*F*_(2,63)_ = 4.49, *p* = 0.015), with lower contour detection performance overall for patients than for healthy controls and first-degree relatives. No significant interaction between group and condition was observed (*F*_(4,126)_ = 1.76, *p* = 0.140).

Following up on the significant main effect of group, we tested for group differences on each condition. Patients performed significantly worse than controls and relatives in the Random condition (Tukey’s HSD, *p* values < 0.01, Cohen’s d = 1.12), significantly worse than relatives in the Orthogonal condition (Tukey’s HSD, *p* = 0.041, Cohen’s d = 0.68), and showed a trend toward poorer Orthogonal performance versus controls that did not survive correction for multiple comparisons (uncorrected *p* = 0.006, Cohen’s d = 0.50). Trends toward poorer contour detection performance among patients in the Parallel condition also did not survive multiple comparisons correction (uncorrected *p* values < 0.035, Cohen’s *d* values > 0.40). Performance did not differ significantly between controls and relatives in any condition.

To explore whether demographic factors were associated with task performance, correlations were examined between contour detection thresholds and demographic values across all groups. We observed significant correlations between both Random and Orthogonal condition thresholds and estimated IQ scores (*r*_(58)_ = 0.54 and 0.49, FDR corrected *p* values < 0.001 and 0.002, respectively). Within groups, there were significant correlations between estimated IQ and Random / Orthogonal thresholds among schizophrenia patients (*r*_(23)_ = 0.70 and 0.68, FDR corrected *p* values < 0.008 and 0.014, respectively), but not for controls or relatives (uncorrected *p* values > 0.306). Other demographic factors (education, CPZ equivalents, BPRS and SPQ scores) were not significantly correlated with contour detection thresholds (FDR corrected *p* values > 0.441). This analysis indicated that baseline task performance may depend in part on IQ score; subsequent analyses therefore focused on effects of context within individuals, since performance differences between flanker conditions should not be confounded by an overall effect of IQ score.

### Contextual Modulation

**Figure 3:**
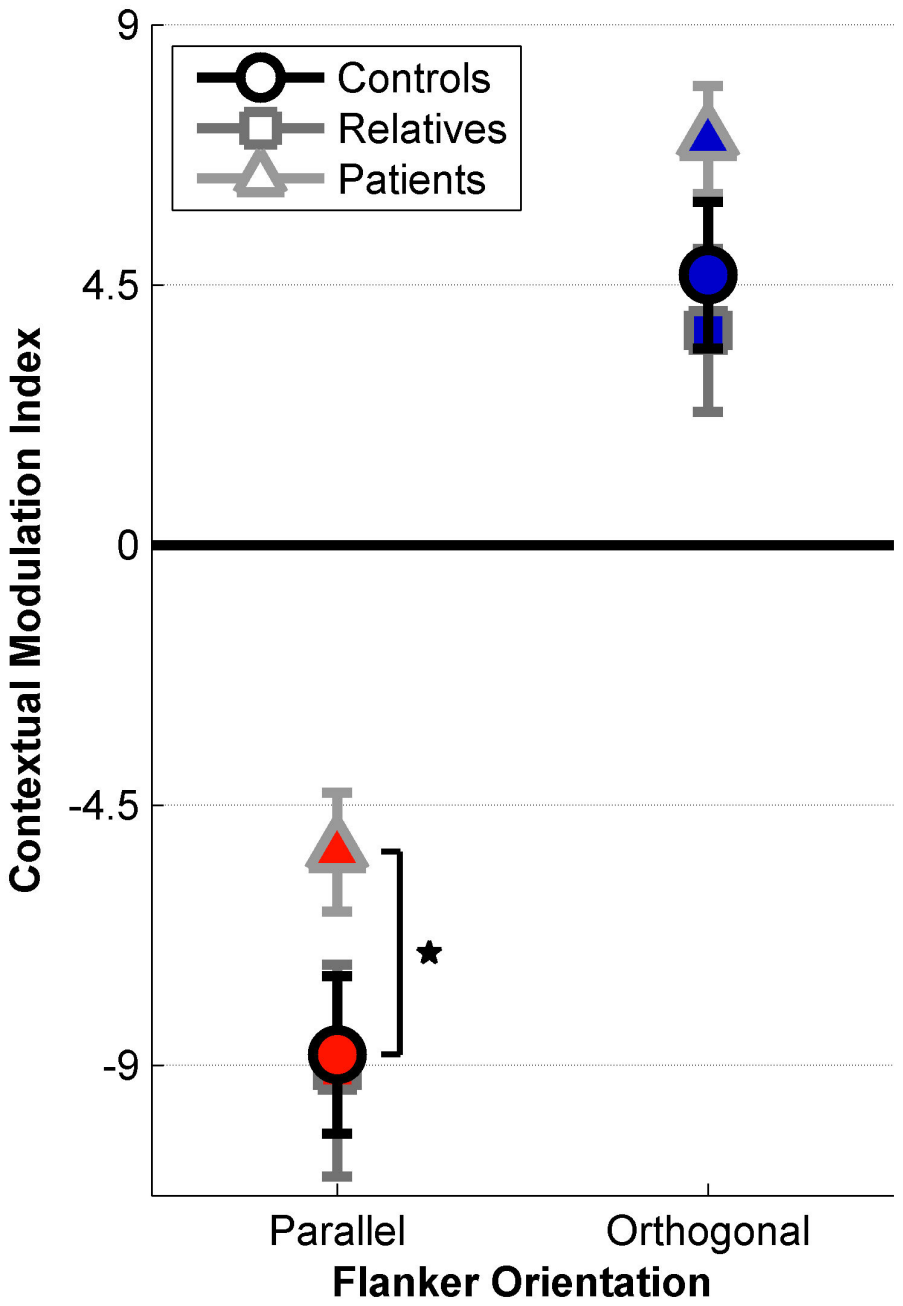
Contextual Modulation Indices. Mean indices are plotted for 28 healthy controls (circles), 13 first-degree relatives (squares), and 25 patients with schizophrenia (triangles) in the Parallel (red) and Orthogonal (blue) conditions. Negative indices indicate conditions where contour detection was suppressed relative to the Random condition, whereas positive indices indicate enhanced contour perception. Asterisk indicates a significant difference between schizophrenia patients and healthy controls, corrected for multiple comparisons via Tukey’s HSD, *p* < 0.05. Error bars are S.E.M.

As patients with schizophrenia show diminished ODSS effects [20], we predicted that the effect of flanker orientation on contour detection performance would be relatively weaker among patients. In order to test this hypothesis, we calculated Parallel and Orthogonal contextual modulation indices for all subjects (see Figure 3). Indices were obtained by subtracting the Random condition threshold from those obtained in the Parallel and Orthogonal conditions. This metric quantified the relative effect of flanker orientation in terms of increased or decreased tolerance to orientation jitter within the contour, irrespective of overall task performance. Patients showed abnormal contextual modulation compared with first-degree biological relatives and healthy controls (ANOVA, *F*_(2,63)_ = 3.15, *p* = 0.049).

Post-hoc analyses revealed that in the Parallel condition, contextual modulation indices were significantly weaker among patients than controls (Tukey’s HSD, *p* = 0.017, Cohen’s d = 0.68). This indicated that patients with schizophrenia could tolerate relatively more orientation jitter within the target contour in the Parallel condition, and thus performed *better* than the controls in the presence of suppressive parallel flankers, relative to the Random condition. There were trends toward higher Orthogonal indices among patients versus controls (uncorrected *p* = 0.044, Cohen’s d = 0.47), and for higher indices among patients versus relatives in both conditions (uncorrected *p* values < 0.02, Cohen’s *d* values > 0.70); however, these did not survive correction for multiple comparisons. Contextual modulation indices did not differ between controls and relatives (uncorrected *p* values > 0.250, Cohen’s *d* values < 0.19). In addition, contextual modulation indices were not significantly correlated with demographic factors (estimated IQ, education, CPZ equivalents, BPRS and SPQ scores, FDR corrected *p* values > 0.321).

### Computational Modeling

**Figure 4:**
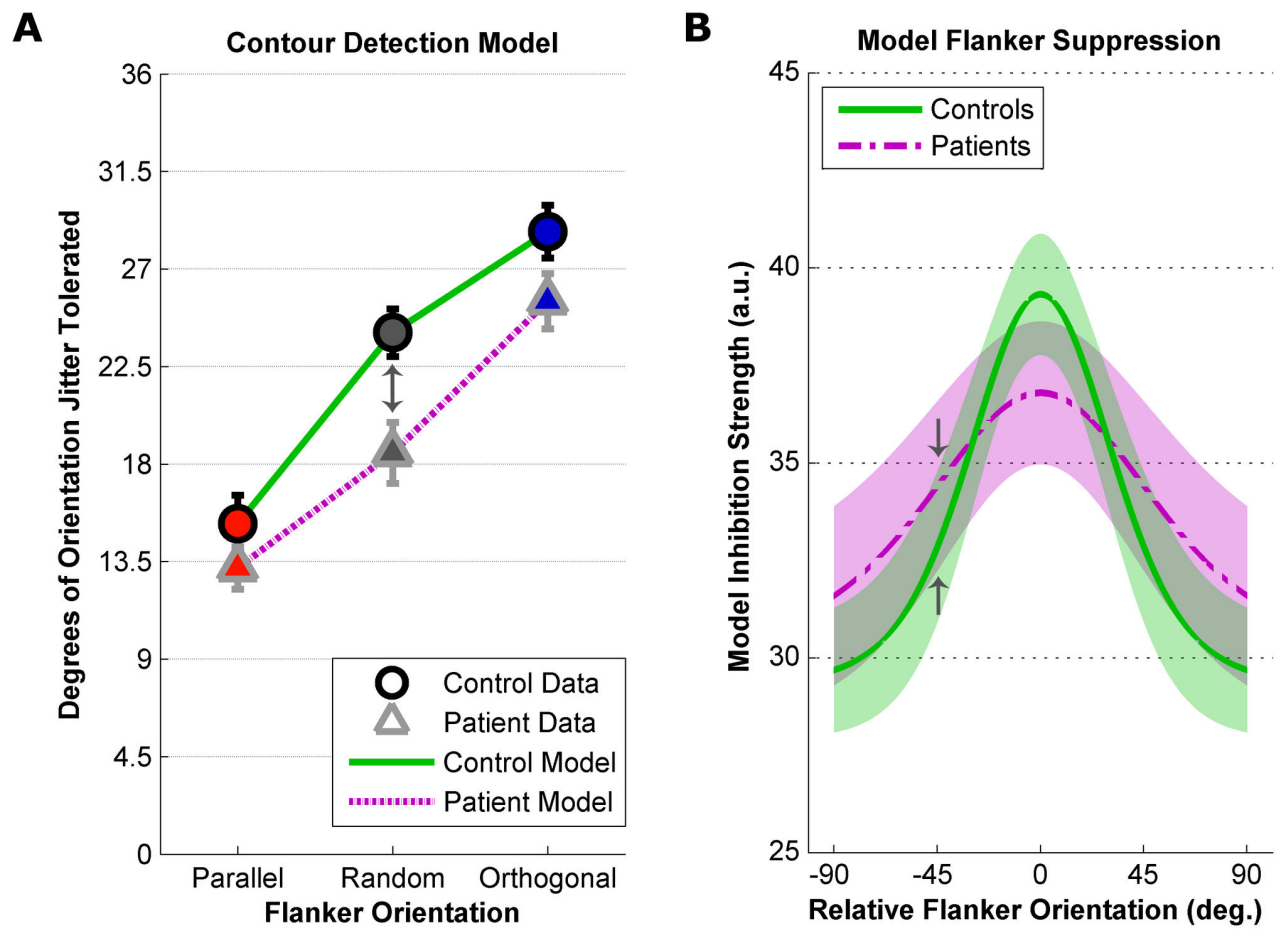
Computational Modeling. Computational model predications based on parameters from Table 2 for 28 control subjects (green solid lines) and 25 schizophrenia patients (purple dashed lines). A) Equation 1 was fit to contour detection task data in all three flanker conditions from healthy controls (circles) and patients with schizophrenia (triangles). Plotted thresholds are identical to those in Figure 2. Error bars are S.E.M. B) Bootstrapped estimates of *T*_0_, *c*_*s*_ and σ_*s*_ were used to calculate flanker suppression orientation tuning distributions (in arbitrary units) for the control and patient groups. Lines plot the mean bootstrapped tuning curves for patients and controls. Shaded regions illustrate 1 standard deviation of the bootstrapped distributions for each group. Gray arrows indicate corresponding positions in both panels.

We fit the contour detection thresholds in all three flanker conditions from patient and control groups with a computational model that allowed us to quantify the effect of relative orientation of flanking elements on contour detection performance (Equation 1, Methods). Figure 4A shows control and patient contour detection thresholds (same data as in Figure 2) with the model predictions from the fit parameters shown in Table 2.

**Table 2:** Computational Model Parameters.

**Parameter:**	*T*_*0*_	*c*_*s*_	*σ*_*s*_
**Values for control subjects:**	29.2 (26.6 : 31.5)	-13.9 (-16.7 : -11.5)	0.54 (0.41 : 0.71)
**Values for schizophrenia patients:**	30.4 (26.0 : 31.5)	-17.2 (-20.0 : -13.0)	0.89 (0.64 : 1.07)

Parameters for computational modeling of contour detection task performance. Parameters were fit to group contour detection thresholds from all three flanker conditions using Equation 1 (see Methods). Values shown are the parameters best fit to the group data (values in parentheses indicate bootstrapped 95% confidence intervals).

Bootstrap analyses showed that model parameters *T*_0_ (baseline contour detection performance) and *c*_*s*_ (overall magnitude of flanker suppression) were not significantly different between groups (*Z* scores = 0.67 and 1.34, FDR corrected *p* values = 0.75 and 0.18, respectively). Values of σ_*s*_, however, differed between groups (*Z* = 3.29, FDR corrected *p* < 0.002), with the model predicting significantly wider orientation tuning of flanker suppression for patients versus controls. Bootstrapped flanker suppression orientation tuning functions for both groups are shown in Figure 4B. These illustrate that for patients, more broadly tuned suppression (vs. controls) fit the data well. In summary, computational modeling of experimental data suggests that flanker suppression, which impairs contour detection accuracy, may be more broadly tuned for orientation among patients with schizophrenia.

## Discussion

We observed impaired contour detection performance among patients with schizophrenia compared with healthy controls and first-degree biological relatives of patients. We also found abnormal contextual modulation among patients, with parallel flankers causing less of a performance decrement (relative to random) for patients than for controls. One explanation for our pattern of results is that schizophrenia leads to an overall impairment in contour integration, in agreement with previous reports [8–13,35–39]. This is supported by the group difference observed in contour detection thresholds (but no significant group by condition interaction), with patients showing poorest group mean performance in all three flanker conditions. An overall deficit in contour perception may be somewhat offset in the Parallel condition by diminished ODSS in schizophrenia [20], as evidenced by reduced Parallel contextual modulation found in post-hoc analyses. These competing effects may have limited the power of our study to detect a group by orientation context interaction, as observed previously [20].

Alternatively, our results may be consistent with less selective orientation detectors in early visual cortex [40], which could give rise to broader tuning of ODSS in schizophrenia. For nearby stimuli within a wide range of flanker orientations, broader ODSS could impair the perceptual salience of a contour during feature integration. We observed the greatest difference in contour detection performance between patients and controls with randomly oriented flankers (which on average are oriented 45° relative to the contour), consistent with the large difference in suppression between groups demonstrated in our model at this flanker orientation (e.g. gray arrows, Figure 4). Thus, our model characterized the pattern of results in terms of broader orientation tuned suppression without significant differences in overall performance or suppression strength. Broader orientation tuning among patients should produce weaker Parallel contextual modulation, but larger Orthogonal modulation compared with controls, indicating greater release from flanker suppression between Random and Orthogonal conditions in schizophrenia. Consistent with this proposal, we observed an overall group difference in contextual modulation (but no significant group by condition interaction), weaker Parallel suppression, and a trend toward greater Orthogonal enhancement for patients. Similarly broad tuning has been reported for basic visual responses among schizophrenia patients [40], and others have proposed that broader tuning for stimulus features may underlie abnormal visual masking in schizophrenia [41].

Although both poorer contour integration with weak flanker suppression and broader orientation tuning with intact contour integration may account for the results we observed in schizophrenia, it is not straightforward to distinguish between these explanations in the current paradigm. One method for teasing apart such proposals would be to examine contextual modulation of contour perception as a function of spacing between stimulus elements. As element separation increases, overall contour detection performance should decrease, but flanker effects should decrease more rapidly, resulting in weak or absent contextual modulation at higher spacing [15]. A recent report examined contour integration in schizophrenia at two spacing levels [35]. They found evidence of a contour integration deficit among patients at target Gabor separations of 0.7° and 1.4° (relative spacing was 3.5λ and 7λ, approximately 2-4 times the contour spacing in the current study). While their study did not manipulate flanker orientation, they did observe a consistent performance deficit across a range of spacing over which our previous work [15] indicates that flanker effects should change dramatically. Their results therefore suggest that broader orientation tuning of flanker suppression cannot fully account for the contour perception deficits observed in the current study among schizophrenia patients. Others have recently reported weaker suppression of contour perception by parallel flankers and poorer orientation discrimination in patients with schizophrenia vs. controls, and interpreted their results within the framework of impaired visual crowding [42].

The current study is the first to investigate whether genetic factors are associated with abnormal contour detection in schizophrenia by examining the performance of patients’ first-degree biological relatives. We observed no significant difference between healthy controls and relatives in either contour detection performance or in contextual modulation. From this, we conclude that abnormal contour perception likely has a closer association with the pathophysiology of schizophrenia, rather than with a genetic liability for this disorder. However, the number of relatives included (*n* = 13) is somewhat low for a psychophysical study in a clinical population, and was smaller than that of our other groups, which may have limited the current study’s power. This smaller sample size may increase the possibility of Type II errors, if for example a large proportion of recruited relatives happen by chance not to be carriers of a genetic variant that influences contour perception, if such genetic factors were to exist. While we find that abnormalities in contour integration and flanker suppression may not prove useful as endophenotypes in schizophrenia, they may instead serve as markers of the current state of neurobiological functioning within the visual system.

Previous work has touched on the role of genetics in other early visual processing tasks in schizophrenia. Our results showing normal behavioral performance among relatives of schizophrenia patients agree with the findings of Silverstein and colleagues [43], who observed that subjects at high risk for developing schizophrenia tended to perform as well as healthy controls in a perceptual organization task. Our findings also agree with previous work showing that symptom remission in patients with disorganized schizophrenia (but not in other psychiatric patients) coincided with improved contour integration [10]. These reports offered preliminary evidence that visual integration deficits in schizophrenia have an association with disease processes, but not genetic factors. By examining contour detection performance among unaffected first-degree biological relatives of schizophrenia patients, we have provided a stronger test of this hypothesis.

Our results agree in part with the findings of Uhlhaas and colleagues [44], who observed poorer contour integration and weaker context modulation of perceived size among non-clinical schizotypal subjects with disordered thoughts. While we did not assess thought disorders in the current study, we found no association between clinical rating scale scores (BPRS and SPQ) and task performance, which may be related to the fact that the recruited outpatients were not highly symptomatic. In contrast to our current results, others have found abnormal backward masking in schizophrenia vs. controls during Vernier discrimination, but no difference between groups in the effect of orientation context [45,46]. Previous work has also shown impairments among unaffected relatives of schizophrenia patients during visual backward masking [47]. It is possible that genetic factors have a stronger influence on the temporal dynamics of early visual processing compared with static pattern vision.

Our task was designed to examine whether contour detection specifically is impaired in schizophrenia. We asked subjects to detect open vertical contours rather than closed figures of a particular shape, thereby mitigating the role of shape representation in task performance. Previous reports of contour integration deficits in schizophrenia have required subjects to locate closed contours at variable positions within the stimulus array [8–12], or to discriminate contour shape configurations [13,35,36]. Such tasks required subjects to identify contours whose features (position within an array, global orientation, and local curvature) varied between trials. Performance within these paradigms relies on a subject’s ability to distinguish the shape of the figure formed by the closed contour. A recent report suggested that perceptual integration deficits in schizophrenia might depend more on impaired shape representation than on abnormal contour detection [35]. The current study did not require visual search or shape discrimination during task performance, yet we nonetheless observed poorer contour detection performance among patients in the Random condition (the one most directly comparable to previous studies). Our results therefore suggest that abnormal contour detection in schizophrenia is a specific perceptual abnormality that is distinct from shape representation impairments that may additionally exist in this disorder.

In the current paradigm, we sought to exclude the role of non-specific deficits among schizophrenia patients. A recent report suggested attentional factors may account for the weak contrast-contrast effect [24] observed when schizophrenia patients reported the perceived contrast of stimuli with and without surrounding context [18] (but see [22]). Our study sought to rule out such potential confounds in the following ways: (1) there is no categorical difference between our stimulus conditions based on the absence of surrounding stimuli [18,24], so equal effort/attention is required in all conditions; (2) we equated difficulty across flanker conditions using a staircase method to manipulate contour jitter, so all subjects performed at the same level (79% correct) in all conditions; (3) we used contextual modulation indices as dependent variables, making the analyses robust against between groups differences in attention or IQ that might impact overall performance. Indeed, we did not observe significant correlations between contextual modulation indices and estimated IQ, despite such correlations being observed between IQ and contour detection thresholds in the Random and Orthogonal conditions. Task performance among patients was also relatively better than for healthy controls in the presence of parallel flankers, which further argues against the notion that a generalized deficit could account for this result. Others have previously demonstrated that perceptual integration abnormalities in schizophrenia exist independent of a generalized deficit, using tasks in which poorer integration leads to a performance advantage among patients [48,49]. Finally, previous reports have demonstrated normal fixation in schizophrenia [50,51], and fixational instability would not affect contextual modulation.

Our findings relate to several outstanding questions that merit further investigation. First, the physiological correlates of abnormal flanker suppression during contour detection in schizophrenia are not yet clear. Through the use of functional MRI (fMRI), abnormal early visual cortical responses during contour integration have been observed among patients [13], suggesting that schizophrenia leads to disruptions in contour perception at the earliest stages of cortical processing. Further, diminished surround suppression effects have been found to correlate with lower γ-aminobutyric acid (GABA) concentrations in schizophrenia, as measured by magnetic resonance spectroscopy (MRS) [52], lending support to the hypothesis that schizophrenia is related to disruptions in GABAergic inhibition [53]. Future investigations employing spatially localized fMRI and GABA MRS in visual cortex could help elucidate the neural mechanisms underlying these perceptual abnormalities.

The relationship between abnormal contextual modulation and other visual deficits in schizophrenia also requires further investigation. Patients with schizophrenia have trouble organizing perceptual information, and some experience visual hallucinations [48,49]. The Gestalt principles of proximity and similarity [7] agree generally with the spatial and orientation specificity of ODSS, which leads us to speculate that abnormal surround suppression may contribute to poorer Gestalt perception in schizophrenia, thereby impairing perceptual grouping. In addition, abnormal electrophysiological activity within visual cortex has been observed in schizophrenia during illusory contour/Gestalt perception [54–56]. One study found that occipital signal abnormalities were associated with visual hallucinations [56]. Abnormal neural synchrony within occipital cortex may therefore be a hallmark of impaired visual feature-binding, which could contribute to both poor contour integration and visual hallucinations in schizophrenia.

## Conclusion

The current study has affirmed previous reports of abnormal contour perception among patients with schizophrenia [8–13,35,36], even with open vertical target contours presented at fixed spatial positions. In addition, we have found that patients are relatively less impaired by the presence of parallel flanking stimuli during contour detection, compared with healthy adults. This agrees with weaker perceptual surround suppression effects reported in schizophrenia [18–22]. Computational modeling of patient data showed that our results are consistent with broader orientation tuning in schizophrenia [40]. We conclude that schizophrenia leads to deficits in contour detection that are consistent with poorer overall integration and weaker parallel flanker suppression, or with broader tuning for visual stimulus orientation. We did not observe any difference between healthy controls and first-degree biological relatives of schizophrenia patients in contour detection performance or in contextual modulation. These visual processing abnormalities are concomitant with the pathophysiology of schizophrenia, but may not be associated with a genetic liability for this disorder. 
