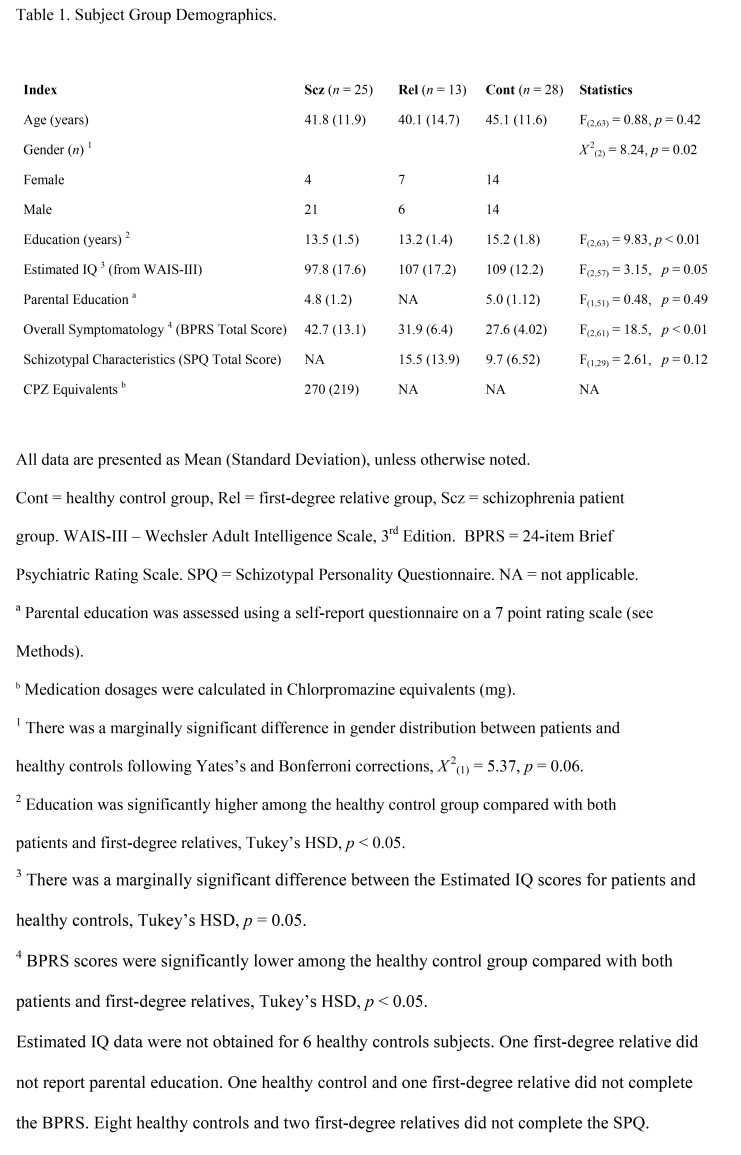# Correction: Abnormal Contextual Modulation of Visual Contour Detection in Patients with Schizophrenia

**DOI:** 10.1371/annotation/f082ec4d-419c-43ce-ae50-e05107539bf3

**Published:** 2013-10-23

**Authors:** Michael-Paul Schallmo, Scott R. Sponheim, Cheryl A. Olman

There was an error in Table 1. The correct version of Table 1 is available here: 

**Figure:**